# GoFundMe as a Medical Plan: Ecological Study of Crowdfunding Insulin Success

**DOI:** 10.2196/33205

**Published:** 2022-04-15

**Authors:** Julia E Blanchette, MJ Tran, Ernest G Grigorian, Eli Iacob, Linda S Edelman, Tamara K Oser, Michelle L Litchman

**Affiliations:** 1 College of Nursing University of Utah Salt Lake City, UT United States; 2 Department of Family Medicine University of Colorado School of Medicine Aurora, CO United States

**Keywords:** diabetes, health care cost, crowdfunding, financial stress, insulin

## Abstract

**Background:**

Individuals in need of medical care turn to crowdfunding websites to engage a “crowd” or group for financial support. In the last decade, access to insulin has decreased considerably for several reasons, including the rising cost of insulin, increasing popularity of high-deductible insurance plans, and increasing insurance premiums. Many people with diabetes are forced to ration or go without insulin, and they turn to crowdfunding websites to seek financial donations to purchase insulin needed to reduce health risks and mortality, and sustain quality of life.

**Objective:**

This study aimed to explore crowdfunding campaign requests to purchase insulin in the United States.

**Methods:**

In this retrospective, quantitative, and qualitative study, we coded the text of GoFundMe online crowdfunding campaigns and viral measures (shares, hearts, and comments) from February 25 to April 15, 2019. We described campaigns (N=205) and explored the factors associated with campaign success using correlations and qualitative thematic analysis.

**Results:**

The majority of campaigns were initiated by middle-aged adults (age 26-64 years; 77/205, 37.6%), those with type 1 diabetes (94/205, 45.9%), and those needing funds owing to insurance coverage issues (125/205, 61.0%). The factors associated with campaign success included requests for ≤US $500 (*P*=.007) and higher viral measures (shares, *P*=.007; hearts, *P*<.001; comments, *P*=.002). The following 4 themes emerged from the campaign text: (1) desire for self-management and survival, (2) diabetes management untenable given insulin access, (3) aftermath of insulin unaffordability, and (4) privacy issues with crowdfunding. Campaign comments were both supportive (tangible, informational, and emotional) and unsupportive (questioned the need for the campaign and deemed crowdfunding inappropriate).

**Conclusions:**

Despite crowdfunding websites being used to support the purchase of insulin, campaigns raised only a fraction of the money requested. Therefore, GoFundMe campaigns are not a reliable solution to obtain funds for insulin in the United States. Applying quantitative and qualitative methods is adequate to analyze online crowdfunding for costs of medications such as insulin. However, it is critical for people with diabetes to use resources other than online crowdfunding to access and obtain insulin owing to low success rates. Clinicians should routinely assess difficulty accessing or affording insulin, and federal health care policies should support lowering the cost of insulin.

## Introduction

All people living with type 1 diabetes and many with type 2 diabetes require insulin to sustain life. People with diabetes are at higher risk for diabetes-related complications and death if they cannot access insulin, even for short intervals. In the last decade, access to insulin has declined considerably due to a myriad of causes, including the rising cost of insulin, increasing health insurance premiums, and increasing popularity of high-deductible insurance plans [1,2].

The price of insulin per unit doubled between 2012 and 2016 [2]. Additionally, close to 9% of the US population was without health insurance in 2017 [3]. Despite Medicaid expansion, which has greatly increased insurance access for lower income adults under the age of 65 years, other insurance barriers hinder insulin affordability. Due to the high cost of insulin, it is difficult for people with diabetes without insurance or with private high-deductible insurance plans to pay for insulin. Cost-related insulin rationing occurs in 1 in 4 people with diabetes and has been associated with detrimental impacts on glycemic outcomes [4]. In addition to health consequences, difficulty affording insulin can contribute to significant financial stress and medical bankruptcy [5].

Insulin access and affordability are critical barriers to preventing acute and long-term diabetes complications, yet people with diabetes report lack of support and resources from health care providers, pharmaceutical companies, insurance companies, hospital systems, and pharmacies [6]. Consequently, some people with diabetes are turning to social media crowdfunding as an attempt to relieve financial stress and obtain insulin. Crowdfunding campaigns aim to raise money for medical care and avoid bankruptcy through websites shared via social networks [7,8]. GoFundMe represents the largest charitable crowdfunding platform and dominates the global medical crowdfunding market [9].

About 8 million Americans have turned to online crowdfunding for medical expenses, and 50 million have reported donating to such campaigns, most commonly in states without Medicaid expansion [10,11]. Much of the current research focuses on the spread of misinformation on crowdfunding sites [12] and campaigns for experimental cures for certain types of cancers [13]. The factors related to successful campaigns for the medical costs of organ transplants include campaigns led by family members or friends rather than the individual in need, longer campaign length, higher funding goals, and greater “hearts” and shares on social media [14]. However, campaigns for people from historically marginalized racial and gender groups are associated with poorer fundraising outcomes [15]. Overall, as few as 8% of campaigns successfully fund the goal amount requested [11,16,17]. Despite low success rates, GoFundMe campaigns remain a popular platform for Americans with various medical needs and costs.

Online crowdfunding has not been well studied in people with diabetes despite its prevalence and the high cost of insulin [18]. More research is necessary to understand the specific rationales for seeking crowdfunding for diabetes care, such as insulin therapy, and to understand if crowdfunding is a successful solution for increasing insulin access [18]. In light of the dramatic rise in insulin cost, this study aims to explore GoFundMe crowdfunding requests for insulin.

## Methods

### Data Sources

The data sources for this ecological study included (1) GoFundMe Campaigns (campaigns), a US-based website for crowdfunding, (2) Face++, a facial recognition (FR) software [19], and (3) the 2017 United States Census. Face++ data were used when age and/or gender were not stated in the campaign. Ideally, we would have collected race from the Face++ software, but race detection was recently removed as a software feature. Data were collected between February 25, 2019, and April 15, 2019. All campaigns included were closed and no longer accepting donations at the time of data collection.

To be included in this study, GoFundMe campaigns had to focus on crowdfunding to purchase insulin for humans, be initiated in the United States given the differences in insurance access, and be written in English. Each campaign website specifies the location, including the country of origin. Campaigns were excluded if they primarily focused on noninsulin diabetes medications, glucometers, glucometer test strips, insulin pumps, or continuous glucose monitors without mentioning insulin, or requested funds for an animal with diabetes. Several search terms were analyzed to determine the search strategy. Given the focus on access to insulin, the term “insulin” and brand names of insulin, including misspellings (ie, Lantus and Lantis) were included in the initial search.

### Ethics Approval

The University of Utah Institutional Review Board acknowledged this study as nonhuman research (#00105240).

### Data Collection Measures

We used Research Electronic Data Capture (REDCap), a web-based study management system [20], to build an online survey for the researchers to extract quantitative and qualitative data about each campaign meeting the study criteria.

#### Age

“Actual age” was extracted from the campaign, when available, and photos were uploaded to the Face++ software if values were missing (“FR age”). It is important to note that not every campaign had a facial photograph of the recipient (eg, landscape and flower). Age was categorized as pediatric (≤17 years), young adult (18-25 years), adult (26-64 years), and older adult (≥65 years). Correlation (*r*) between actual age and FR age was 0.395 (*P*=.003). Face++ detected the age group for 87 of the 139 missing data cases or 42.4% of the total sample. Overall, 52 (25%) campaigns had missing photos or undetectable age by Face++.

#### Gender

“Actual gender” was extracted from the campaign by coding pronouns. Campaigns were coded as male if the individual requiring insulin was referenced as he, him, his, dad, brother, uncle, or grandfather. Campaigns were coded as female if the individual requiring insulin was referenced as she, her, hers, mom, sister, aunt, or grandmother. Campaigns were coded as nonbinary if the individual requiring insulin was referenced as they or them. Face++ facial detection software was used to code campaign photos as male or female (“FR gender”) when gender was not available in the text. The correlation (*r*) between actual gender and FR gender was 0.926 (*P*<.001). Face++ detected gender for 31 of the 68 missing data cases or 15% of the total sample. Overall, 37 (18%) campaigns had missing photos or undetectable gender by Face++.

#### Flesch-Kincaid Education

A Flesch-Kincaid score was identified to understand the education level in which the campaign was written. Scores were analyzed as a continuous variable (grade 0 to ≥13).

#### Income

Based on the city and state where the campaign originated, the city- and state-level median income and percentage of residents at the poverty level were extracted from the 2017 US Census.

#### Geographic Designation

Based on the city where the campaign originated, city population size was extracted from the 2017 US Census. County of residence was determined for each campaign and assigned a Rural-Urban Continuum Code (RUCC), and the codes were then collapsed into metro (RUCC codes 1-3), urban (RUCC codes 4-7), and rural (RUCC codes 8-9) categories [21].

#### Insurance Status

Based on the state where the campaign originated, state Medicaid expansion status (yes/no) and the percentage of the state uninsured population were extracted from the 2017 US Census.

#### Financial Information

The amount of funds requested, amount of funds raised, time (months) the account was active, and number of funders were extracted from the campaigns.

#### Viral Information

The number of shares on Facebook and Twitter combined and number of hearts on the GoFundMe website were extracted from the campaigns.

#### Campaign Initiator

Information about the campaign requestor was extracted from the campaign, including the relationship to the people with diabetes requiring insulin (self, friend or family, and other) and geographic location (city and state).

#### Rationale for Request

The campaigns were coded for the following rationales: uninsured or inadequate insurance, change in personal finance, personal emergency, general fundraising, or other. Multiple categories could be selected. The brand name of insulin, when mentioned, was coded.

#### Qualitative Data

The entire text of the campaign and the associated comments were extracted separately.

### Statistical Analysis

Data were exported into SPSS (IBM Corp) for analysis. Since multiple search terms were used to identify the campaigns, duplicate campaigns were removed before analysis. A total of 44 duplicate campaigns were removed before data analysis. One outlier for the number of funds requested (US $1,000,000.00) was removed due to the extreme amount.

Descriptive statistics, including frequencies, were tabulated. Missing data were handled pairwise. The following research questions (RQs) guided the analysis: (1) Who started the campaign? (2) What was the purpose of the campaign? (3) What was the success of the campaign? (4) What factors were associated with campaign success?

A qualitative content analysis of campaign posts and comments was conducted for RQ1 and RQ2. Two independent researchers read textual data, line by line, and coded the data using an open code approach [22,23]. Codes were used to organize similar data to identify the rationale for the campaign and commenter responses [22]. A matrix of the types of support offered by commenters was developed. A third author facilitated consensus to establish credibility. Given the sensitivity of the topic and the fact that campaign requestors developed some campaigns without the knowledge of people with diabetes, no direct quotes were used in this manuscript to protect possible identification. Student *t* tests and Fisher exact tests were used for RQ3 and RQ4 to describe the factors associated with campaign success rates. Fisher exact tests were used for associations due to categorical data and small frequencies in the fully funded categories.

## Results

### Sample

A total of 1623 campaigns were reviewed, and 249 met the inclusion criteria. After removing 44 duplicates, a total of 205 GoFundMe campaigns were included in the final analysis.

The Face++ software could not predict age and gender when the photograph quality was poor or when the campaign did not include a photograph. Age and gender predictions from Face++ software were highly correlated with age (*r*=0.395; *P*=.003) and gender (*r*=0.926; *P*<.001) stated in the campaigns, when available.

**Figure 1:**
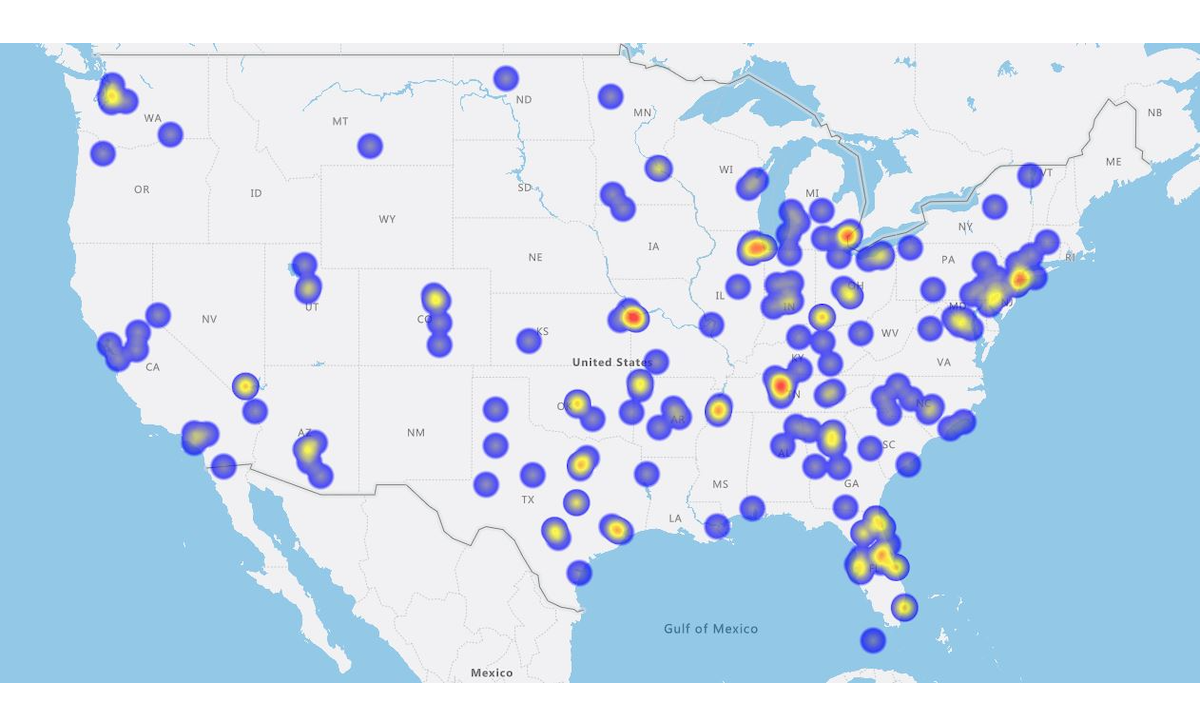
Geographic heat map of GoFundMe campaigns.

Campaigns for people residing in the southern United States (100/205, 48.8%) and in metro geographic locations (176/205, 85.9%) were the most frequent. Table 1 provides demographic characteristics, and Table 2 provides diabetes-specific and campaign characteristics. Figure 1 provides a geographic heatmap of campaigns.

**Table 1:** Sample demographic characteristics with the Fisher exact test to examine contributors to the funding status (N=205, unless otherwise specified).

Variable		Fully funded (n=22), n (%)	Not funded (n=183), n (%)	Total (N=205), n (%)	*P* (Fisher exact test)
**Requestor**					N/A^a,b^
	Self	9 (41)	105 (57)	114 (56)	
	Family or friend	10 (46)	62 (34)	72 (35)	
	Other	3 (14)	16 (9)	19 (9)	
**Gender^c^**					N/A
	Male	13 (59)	63 (34)	76 (37)	
	Female	5 (23)	87 (48)	92 (45)	
	Unable to determine (no photo/poor quality photo)	4 (18)	33 (18)	37 (18)	
**Age group^c^**					N/A
	Pediatric (<18 years)	0 (0)	12 (7)	12 (6)	
	Emerging adult (18-25 years)	1 (5)	35 (19)	36 (18)	
	Middle adult (26-64 years)	9 (41)	68 (37)	77 (38)	
	Older adult (≥65 years)	6 (27)	22 (12)	28 (14)	
	Unable to determine (no photo/poor quality photo)	6 (27)	46 (25)	52 (25)	
**US region**					N/A
	West	5 (23)	30 (16)	35 (17)	
	Midwest	7 (32)	45 (25)	52 (25)	
	Northeast	2 (9)	16 (9)	18 (9)	
	South	8 (36)	92 (50)	100 (49)	
**Medicaid expansion state**					.82
	Yes	13 (59)	101 (55)	114 (56)	
	No	9 (41)	82 (45)	91 (44)	
**Flesch-Kincaid education score**					.82
	≤8	13 (59)	113 (62)	126 (62)	
	9+	9 (41)	70 (38)	79 (39)	

^a^N/A: not applicable.

^b^Did not analyze the data with the Fisher exact test owing to more than 20% missing data.

^c^Face++ facial recognition software was used to determine the approximate age and gender of GoFundMe recipients when age and gender were not available.

**Table 2:** Campaign and diabetes-specific characteristics with the Fisher exact test to examine contributors to the funding status (N=205, unless otherwise specified).

Variable			Fully funded (n=22), n (%)		Not funded (n=183), n (%)		Total (N=205), n (%)		*P* (Fisher exact test)
**Type of diabetes**									N/A^a,b^
	Type 1 or latent autoimmune diabetes in adults	10 (46)		84 (46)		94 (46)			
	Type 2	2 (9)		17 (9)		19 (9)			
	Gestational	0 (0)		2 (1)		2 (0)			
	Unknown	10 (46)		80 (44)		90 (44)			
**Reason for request**									N/A
	Insurance/system issue (uninsured, underinsured)	12 (55)		113 (62)		125 (61)			
	Personal issue (loss of job, emergency)	1 (5)		10 (6)		11 (5)			
	General fundraiser	8 (26)		41 (22)		49 (24)			
	Insurance/system and personal issue	1 (5)		19 (10)		20 (10)			
**Insulin type^c^**									N/A
	Fast-acting (Apidra, Admelog, Fiasp, Afrezza)	2 (9)		50 (23)		52 (21)			
	Long-acting (Lantus, Levemir, Basaglar, Toujeo, Tresiba)	2 (9)		39 (18)		41 (17)			
	Intermediate/mixed/regular (neutral protamine hagedorn insulin [NPH], insulin regular human [R])	0 (0)		15 (7)		15 (6)			
	Concentrated (Humalog U200, U500)	1 (4)		3 (1)		4 (2)			
	Other	0 (0)		1 (1)		1 (0)			
	Unknown	18 (78)		114 (51)		132 (54)			
**Insulin requests**									N/A
	One or more types requested	4 (18)		69 (38)		73 (36)			
	Unknown	18 (81)		114 (62)		132 (64)			
**Medicare donut**									.74
	Yes	3 (14)		22 (12)		25 (12)			
	No or not mentioned	19 (86)		161 (88)		180 (88)			
**Disability status**									>.99
	Yes	2 (9)		16 (9)		18 (9)			
	No or not mentioned	20 (91)		167 (91)		187 (91)			
**Pharma support^d^**									N/A
	Requested and rejected	1 (5)		9 (5)		10 (5)			
	Not mentioned (unknown)	21 (96)		174 (95)		19 (95)			
**Request amount**									.007
	≤US $500	9 (41)		28 (15)		22 (11)			
	>US $500	13 (59)		155 (85)		183 (89)			
**Funding length**									.82
	<3 months	7 (32)		68 (37)		75 (37)			
	≥3 months	15 (68)		116 (63)		130 (63)			

^a^N/A: not applicable.

^b^Did not analyze with the Fisher exact test owing to more than 20% missing data.

^c^For this variable, the N values for fully funded, not funded, and total were 23, 222, and 245, respectively.

^d^N is >205, as some requests were more than one.

### Income Descriptive

Campaign requestors originated from cities and states with median poverty levels of 17.36% (range 0.70%-47.20%) and 14.99% (range 9.70%-21.50%), respectively, based on the 2019 United States Census. The median household incomes in the cities and states of the requestors were US $52,639.86 (range US $27,838-$124,922) and US $55,537.48 (range US $42,009-$78,916), respectively, which were below the 2019 national average income of US $68,703.

### Viral Measures

Campaigns received support through several donors (median 9, range 0-116) and shares (median 9, range 0-742). However, campaigns had a median of 0 (range 0-20) comments and a median of 2 (range 0-70) hearts.

### RQ1: Who Started the Campaign?

The campaign requestor was commonly the person with diabetes in need of funds (114/205, 55.6%), followed by family members (46/205, 22.4%). Friends (26/205, 12.7%) less commonly requested funds. Requests were most frequently for people in the age group of 26-64 years (80/205, 39.0%), with the majority who specified diabetes type (n=115) having type 1 diabetes (94/115, 81.7%).

### RQ2: What Was the Purpose of the Campaign?

About half of the campaigns (99/205, 48.3%) described how long the funds would last. Of those, 29% (29/99) needed quick funds to cover cost needs for <3 months, while 71% (70/99) needed funds that would last ≥3 months.

The most common campaign purpose was to fund insulin for people with diabetes having no insurance or inadequate insurance coverage, or an insurance system issue (125/205, 61.0%), followed by general fundraising (49/205, 23.9%), personal and insurance issues (20/205, 9.8%), and personal issues (loss of job or emergency) (11/205, 5.4%).

Just under half of the requestors lived in non-Medicaid expansion states (91/205, 44.4%). Cost issues related to the Medicare gap were reported in 12.2% (25/205) of campaigns.

A total of 245 insulin requests were made as some people with diabetes used 2 types of insulin. Fast-acting insulin (Novolog, Humalog U100, Apidra, Admelog, Fiasp, and Afrezza) was the most commonly requested (52/245, 21.2%), followed by long-acting insulin (Lantus, Levemir, Basaglar, Toujeo, and Tresiba) (41/245, 16.7%). The remaining types of insulin requested were intermediate-acting, mixed, or regular insulin (15/245, 6.1%), or concentrated insulin (4/245, 1.6%). Most insulin types (132/245, 53.9%) were not specified.

### RQ3: What Was the Success of the Campaign?

Campaign goals ranged from US $50 to US $200,000 (median US $1100), while the amount raised ranged from US $0 to US $6920 (median US $65). Over one-third (77/205, 37.6%) of campaigns raised US $0, while just over 10% (22/205, 10.7%) of campaigns were fully funded. The top quartile of campaigns raised only 33.4% of the requested funds, although the range of funding was 0% to 583% (median 4%).

### RQ4: What Factors Were Associated With Campaign Success?

The amount of money raised correlated with all viral measures, including the number of shares (median 9, range 0-742; *U*=1319.50; *P*=.007), number of hearts (median 2, range 0-70; *U*=614.50; *P*<.001), and number of comments (median 0, range 0-20; *U*=1061; *P*=.002). Factors, including Medicaid expansion state, Flesch-Kincaid education, Medicare donut hole status, disability status, and funding length, were not significantly associated with success in raising funds. Requests ≤US $500 were more likely to receive funding (Fisher exact *P*=.007). See Tables 1 and 2 for more information.

### Qualitative Analysis of Campaign Posts

Campaign requestors described a myriad of issues surrounding the cost of insulin and privacy issues related to crowdfunding in general.

#### Desire for Self-management and Survival

Campaigns were often started because people with diabetes actively wanted to participate in diabetes self-management, yet lacked the funds. Themes included wanting to manage diabetes to be healthy enough to care for young children and contributing to society by continuing effective diabetes self-management that is critical to being a productive employee.

Campaign requestors emphasized that obtaining funds to afford insulin was the key to avoiding hospitalization and described how an emergency room visit or inpatient hospital stay would only exacerbate costs for those already struggling to afford insulin.

Alongside avoiding hospitalization, campaign requestors emphasized their desire to live and prevent premature death. Some campaign requestors narrated who they wanted to live for (emphasizing family), why their life mattered (how they contribute to society), what they wanted to continue doing with their lives (work, hobbies, caretaking, etc), and how insulin was necessary to avoid death.

As there are different types of insulin on the market, some campaign requestors overtly rationalized their need for brand-name insulin. Examples of needing brand-name insulin most often focused on a better biophysical response to brand-name insulin than generic insulin. Not all campaigns rationalized why brand-name insulin was desired.

#### Lack of Insulin Access Makes Diabetes Management Untenable

Insulin access issues were described regardless of insurance status. Some people with diabetes were waiting for new insurance to initiate. Others had recently lost a job and insurance benefits, aged out of Medicaid and were without parent insurance coverage before the age of 26 years, or were disabled and waiting for disability insurance to initiate. Those who described being underinsured included those experiencing the Medicare gap coverage (“donut hole”) or a coverage gap in which there is a temporary limit on what the insurance plan covers after a certain amount of medication costs have already been paid for in a given year. Some with coverage described “fighting” or “going to battle” with insurance about insulin costs without success. Campaign requestors described applying for various discount or financial assistance programs and being denied or not given enough money.

Some campaign requestors described a new diabetes diagnosis after hospitalization. In few cases, the campaigns were developed before the people with diabetes were discharged from the hospital. The sudden expense of hospitalization, in addition to a new or ongoing insulin expense, was overwhelming and financially challenging. The people with diabetes were discharged without a way to cover insulin costs and were fearful they would be readmitted to the hospital.

At times, campaign requestors mentioned that insulin competed with other financial interests, such as addressing personal emergencies (flood in the basement of their home, broken down car, etc) and basic expenses (rent, food, and utilities). In few instances, family member health expenses for conditions, such as cancer, drained family finances and left no money for insulin. Additionally, some people with diabetes in single-income households reported decreased access to resources in general.

Some people with diabetes reported that a specific brand of insulin was more effective, yet insurance only covered an alternative brand. Other people with diabetes described difficulty managing blood glucose when using generic insulin (regular and neutral protamine hagedorn) compared with brand-name insulin. Conversely, some individuals used generic insulin but still could not afford it.

#### The Aftermath of Insulin Unaffordability

When people with diabetes could not afford insulin, the campaign requestors described rationing insulin doses and/or food to avoid diabetic ketoacidosis and fear of dying. Some people with diabetes reported feeling too sickly to attend work or school due to hyperglycemia from insulin rationing.

Some people with diabetes who could not afford insulin went to the emergency room to treat hyperglycemia as a quick solution, leading to additional health care expenses, despite obtaining no-cost insulin coupons or insulin from that visit. Other people with diabetes described alternative ways to access insulin, such as engaging in online insulin trading and seeking insulin donations. In one case, a person with diabetes described having an “insulin dealer” who provided insulin at a cheaper cost than when using insurance.

#### Privacy Issues With Crowdfunding

Many people with diabetes described feeling embarrassed and desperate for resorting to GoFundMe to support their health costs. Campaign requestors who were family members or friends expressed feeling self-conscious or awkward about putting their loved ones with diabetes in the spotlight to get assistance for them.

### Qualitative Analysis of Comments

The majority of campaigns (125/205, 61.0%) had 0 comments, followed by 1-3 comments (58/205, 28.3%) and ≥4 comments (22/205, 10.7%). There were 191 comments across campaigns that were supportive and unsupportive. Supportive comments provided tangible, informational, or emotional support. Unsupportive comments questioned the need for the campaign or stated that the campaign was inappropriate. Table 3 provides examples of comments.

**Table 3:** Types of comments provided by commenters.

Comment type		Examples of comments provided by commenters
**Supportive**		
	Tangible support	Described how much financial support they contributed to the campaign Offered to donate insulin vials/pens via mail or meeting with the people with diabetes Described how they knew the campaign requestor and suggested that others within their social network donate as well
	Informational support	Provided information about where nonanalog generic insulin could be purchased (ie, Walmart for approximately US $25/vial) Provided links to websites with insulin assistance options (ie, coupons or patient assistance programs) Recommended that people with diabetes reach out to health care providers for insulin samples
	Emotional support	Provided well wishes for campaign success Offered prayers for the people with diabetes Described they were people with diabetes and understood what people with diabetes in need were going through
**Unsupportive**		
	Questioned need	Questioned the financial need for a campaign noting that the people with diabetes could afford insulin Raised concern that the campaign was a “scam” (note that in some instances, the campaign requestor would reply with a photo of the diabetes supplies to indicate there was an actual need)
	Crowdfunding is inappropriate	Described GoFundMe as an inappropriate avenue for financial support for diabetes self-management needs

## Discussion

### Principal Findings

To our knowledge, this is the first study to examine crowdfunding requests for insulin. The high cost of insulin places a significant burden on people with diabetes and their supporters, who seek crowdfunding as a solution to raise funds to purchase insulin. We found that the overwhelming majority of campaigns were not fully funded.

About half of the campaigns originated from the southern United States in our study. While it is possible that crowdfunding is more prevalent in southern states, 44.4% of campaigns were started in states without Medicaid expansion. Though there is evidence that the cost of insulin affects all age groups [4], we found that over half of the campaigns were developed for individuals in middle adulthood. Middle adulthood is a time of the greatest financial stability, yet living with diabetes can greatly impact finances, marriage/divorce, raising and launching children, living with bad credit, employment, or medical insurance coverage, or result in exiting the workforce [6,24]. Additionally, those in middle adulthood are less likely to qualify for federal or state insurance programs compared with children and older adults.

Many people with diabetes are desperately trying to identify alternative ways to access insulin owing to its current cost. As identified by other researchers [6], our findings highlight the desire for engaging in diabetes self-management and emphasize the need to avoid hospitalization and prevent additional health care debt. The few fully funded campaigns were associated with requesting ≤US $500. These findings differ from those of previous studies showing that higher funding amounts resulted in greater campaign success. However, previous studies highlighted campaigns for major medical procedures, such as organ transplantation, or costly cancer treatments. As insulin is a life-long and ongoing cost, smaller requests ≤US $500 were likely for 1 month or less of insulin supply. In the context of the total cost of insulin, these small funds may only serve as a “band-aid” to the exorbitant costs endured by insulin users.

The majority of requests were for brand-name insulin. While it was clear some people with diabetes in this study knew they would not respond well to generic insulin, others may have been unaware of the generic insulin option. Some commenters offered informational support about the cost of generic insulin and tangible support via insulin donations. Recent research indicates that some people with diabetes engage in the underground exchange of diabetes medications and supplies with online strangers, including insulin donations [6,25].

Our findings indicate that viral measures correlated with money raised by the campaign. Others have found that successful crowdfunding campaigns leverage collective endorsements through close online networks [6,26], though strangers also donate [27]. Close networks may feel social pressure to donate, even when in a position where they cannot afford to contribute [27]. Individuals without close online networks or those who are digitally and/or linguistically illiterate contribute to a rise in health care disparities [11]. Importantly, campaign requestors and donors may not understand that fees from donations are deducted or understand the validation process required to receive funds.

Though crowdfunding can temporarily increase access to insulin, ethical issues related to crowdfunding for diabetes care exist [28-30]. For example, crowdfunding websites encourage photos, videos, and ongoing updates, resulting in loss of privacy. Although recent evidence suggests that some campaign requestors weigh the need for financial support over the need for privacy [31], we found that nearly one-fourth of campaign requestors were family members or friends. As such, people with diabetes may not be aware of or control what information is shared about them. There is also the possibility of phony crowdfunding accounts to solicit funds.

### Limitations

This study must be interpreted in the context of its limitations. Due to the public nature of the content, some data were limited. We were unable to gather specific clinical characteristics, such as HbA_1c_ and hospitalizations. We were also unable to code insulin pump status, which could influence the number of insulins requested. We also encountered some missing data, such as age and gender. While we could impute age and gender when missing among those with facial photographs using FR software, there were limitations to imputing age. The correlation between provided age and Face++ recognition was only moderate, and due to missing information, we used Face++ age for about 40% of the sample. As insurance coverage and financial stressors vary by age group, it was essential for us to provide age group. However, age group was provided for descriptive purposes only and not used in further statistical analysis. Another limitation of Face++ imputation was the inability to identify race and ethnicity. Finally, we were only able to analyze active campaigns and therefore were unaware if individuals repeatedly started new campaigns.

### Conclusions

Applying quantitative and qualitative methods to analyze online GoFundMe campaigns is effective for understanding success in online crowdfunding for health care and medication costs such as insulin. As purchasing insulin is untenable to many people with diabetes owing to its high cost, crowdfunding through websites, such as GoFundMe, may raise a small amount of money to work as a temporary solution for purchasing insulin, but may not be considered a reliable resource to purchase insulin in the long term. Clinicians must ask people with diabetes if they have difficulty accessing or affording insulin and provide resources at appointments. Additionally, it is essential to focus on solutions, such as health care reform and health care policies, that support lowering the cost of insulin, particularly at the federal level. Hence, all people with diabetes who use insulin should have access to their life-sustaining medication.

## Abbreviations

FR: facial recognition
RQ: research question
RUCC: Rural-Urban Continuum Code
